# Activation of brain‐derived neurotrophic factor signaling in the basal forebrain reverses acute sleep deprivation‐induced fear memory impairments

**DOI:** 10.1002/brb3.1592

**Published:** 2020-03-10

**Authors:** Tao Ma, Hao Zhang, Zhi‐Peng Xu, Yan Lu, Qiang Fu, Wei Wang, Guan‐Hua Li, Ying‐Ying Wang, Yi‐Tian Yang, Wei‐Dong Mi

**Affiliations:** ^1^ Anesthesia and Operation Center Chinese PLA Medical School Beijing China; ^2^ Department of Anesthesiology PLA Rocket Force Characteristic Medical Center Beijing China; ^3^ Department of Neurology PLA Rocket Force Characteristic Medical Center Beijing China; ^4^ PLA Rocket Force Characteristic Medical Center Postgraduate Training Base of Jinzhou Medical University Beijing China

**Keywords:** basal forebrain, brain‐derived neurotrophic factor, fear memory, sleep deprivation

## Abstract

**Introduction:**

The mechanisms underlying sleep deprivation‐induced memory impairments and relevant compensatory signaling pathways remain elusive. We tested the hypothesis that increased brain‐derived neurotrophic factor (BDNF) expression in the basal forebrain following acute sleep deprivation was a compensatory mechanism to maintain fear memory performance.

**Methods:**

Adult male Wistar rats were deprived of 6‐hr total sleep from the beginning of the light cycle. The effects of sleep deprivation on BDNF protein expression and activation of downstream tropomyosin receptor kinase B (TrkB)/phospholipase C‐γ1 (PLCγ1) signaling in the basal forebrain and fear memory consolidation were examined. BDNF or selective downstream TrkB receptor antagonist ANA‐12 was further injected into the basal forebrain bilaterally to observe the changes in fear memory consolidation in response to modulation of the BDNF/TrkB signaling.

**Results:**

Six hours of sleep deprivation‐induced both short‐ and long‐term fear memory impairments. Increased BDNF protein expression and TrkB and PLCγ1 phosphorylation in the basal forebrain were observed after sleep deprivation. Microinjection of BDNF into the basal forebrain partly reversed fear memory deficits caused by sleep deprivation, which were accompanied by increased BDNF protein levels and TrkB/PLCγ1 activation. After ANA‐12 microinjection, sleep deprivation‐induced activation of the BDNF/TrkB pathway was inhibited and impairments of fear memory consolidation were further aggravated.

**Conclusions:**

Acute sleep deprivation induces compensatory increase of BDNF expression in the basal forebrain. Microinjection of BDNF into the basal forebrain mitigates the fear memory impairments caused by sleep deprivation by activating TrkB/PLCγ1 signaling.

## Graphical abstract

We found that acute sleep deprivation could increase BDNF protein expression and downstream TrkB/PLCγ1 activation robustly in the basal forebrain area. Microinjection of BDNF into basal forebrain could further activate TrkB/PLCγ1 signaling and reverse the decline in fear memory consolidation. Moreover, microinjection of TrkB receptor antagonist ANA‐12 could aggravate sleep deprivation‐induced memory impairments.

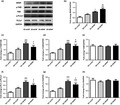

## 1 INTRODUCTION

Sleep deprivation is becoming more prevalent due to contemporary lifestyle and work‐related factors (Bixler, 2009; Dinges, 1995). Nearly 30% of full‐time workers sleep <6 hr a day (CDC, 2005; Knutson, Van Cauter, Rathouz, DeLeire, & Lauderdale, 2010). Sleep deprivation significantly impaired short‐ and long‐term memory in animal and human models (Alvarenga et al., 2008; Havekes, Meerlo, & Abel, 2015; Kreutzmann, Havekes, Abel, & Meerlo, 2015; Li, Yu, & Guo, 2009; Prince et al., 2014; Stickgold, 2005; Vecsey, Park, Khatib, & Abel, 2015). However, the underlying molecular mechanism and brain circuits involved remain elusive.

Within the basal forebrain, a region implicated in sleep–wake control, there are three major types of neurons: cholinergic, glutamatergic, and GABAergic neurons. Research has found that cholinergic neurons are active during wakefulness and rapid eye movement (REM) sleep but remain silent during nonrapid eye movement (NREM) sleep (Knox, 2016; Lee, Hassani, Alonso, & Jones, 2005). Furthermore, the activation of cholinergic neurons can enhance arousal, attention, and memory (Eggermann, Kremer, Crochet, & Petersen, 2014; Everitt & Robbins, 1997; Fu et al., 2014; Jones, 1993; Sarter, Hasselmo, Bruno, & Givens, 2005). Impairment of spatial memory was found to be related to injury of cholinergic neurons and hypofunction of GABAergic neurons in the basal forebrain (Jeong, Chang, Hwang, Lee, & Chang, 2011). The glutamatergic and GABAergic neurons were also found to regulate the transition of cortical activity and sleep–wake states (Fuller, Sherman, Pedersen, Saper, & Lu, 2011; Kroeger et al., 2017). As regards the neural circuit, the basal forebrain is involved in nearly all types of memories by interconnecting the hippocampal and septo–hippocampal circuits, such as fear memory and spatial reference memory (Blake & Boccia, 2018; Givens, Williams, & Gill, 2000; Hall, Gomez‐Pinilla, & Savage, 2018). Nevertheless, it is still unclear whether the basal forebrain is involved in memory impairment caused by acute sleep deprivation.

Of interest, brain‐derived neurotrophic factor (BDNF) emerges as a crucial protein in the regulation of both sleep and memory (Alzoubi, Khabour, Salah, & Abu Rashid, 2013; Bachmann et al., 2012; Datta, Siwek, & Huang, 2009; Giacobbo et al., 2016; Mascetti et al., 2013). It can bind to and activate tropomyosin receptor kinase B (TrkB) receptors, thereby regulating the activity of phospholipase C‐γ1 (PLCγ1) pathway (Hyungju & Mu‐Ming, 2013; Zhao & Levine, 2014). Previous research found that activation of the BDNF/TrkB pathway could improve sleep and memory (Bekinschtein, Cammarota, & Medina, 2014; Monteiro et al., 2017). Intracerebroventricular injection of BDNF could activate BDNF/TrkB signaling, increase NREM sleep in rats, and increase both NREM and REM sleep in rabbits (Kushikata, Fang, & Krueger, 1999; Watson, Henson, Dorsey, & Frank, 2015). However, whether basal forebrain BDNF/TrkB pathway is involved in memory impairment followed by acute sleep deprivation is not clear (Watson, 2015).

We proposed that increased BDNF expression in the basal forebrain following acute sleep deprivation acted as a compensatory mechanism to maintain fear memory performance and conducted this study.

## 2 MATERIALS AND METHODS

### 2.1 Animals

Adult male Wistar rats weighing 280 to 320 grams (*n* = 120; purchased from 301 Experimental Animal Center of PLA Medical College) were used in this experiment. The animals were housed in pairs under a 12‐hr/12‐hr light–dark cycle (lights on at 08:00 AM) in a temperature‐controlled (23 ± 1°C) animal colony room with free access to food and water.

### 2.2 Ethical statement

All procedures involving animals were approved by the Ethics Committee of the Chinese PLA Medical School and were performed in accordance with the Guide for the Care and Use of Laboratory Animals.

### 2.3 Acute sleep deprivation

The gentle handling‐induced sleep deprivation protocol (Oonk, Krueger, & Davis, 2016) was followed. This procedure of sleep deprivation was found to have little effect on stress response. Sleep deprivation started at 8:00 a.m. and lasted for 6 hr. During the procedure, the rats could behave naturally and had free access to food and water. When the rats closed their eyes or stopped whisking, perturbations were used to keep them awake. The perturbation strategies included a soft writing brush to stir the bedding and a finger snap to make noise and slightly tapping or rotating the cage. If the methods above were not effective, rats were touched with the writing brush. No novel objects were used.

### 2.4 Experimental design

#### 2.4.1 Experiment 1

**Figure 1:**
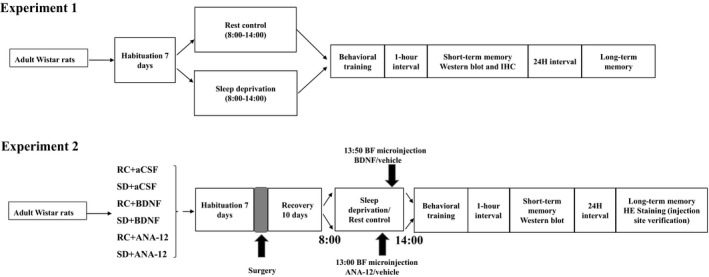
Experimental design schematic. The study consisted of two experiments. Experiment 1 involved assessment of acute 6‐hr sleep deprivation‐induced short‐term fear memory (STM, 1 hr after training) and long‐term fear memory (LTM, 24 hr after training) impairments. The rats were divided into the rest control (RC) and sleep deprived (*SD*) groups. The effects of sleep deprivation on basal forebrain BDNF protein expression, and TrkB/PLCγ1 phosphorylation were also evaluated 1 hr after behavior training. In Experiment 2, exogenous BDNF, TrkB receptor antagonist ANA‐12, or the vehicle control was infused before fear memory training to test the modulating effects of BDNF/TrkB signaling on sleep deprivation‐induced fear memory impairments. BDNF (250 ng/1 μl/side) was infused 10 min before fear memory training, and ANA‐12 (0.5 μg/1 μl/side) was injected 1 hr before fear memory training. After LTM evaluation was finished, the rat brain was collected and subjected to hematoxylin and eosin (HE) staining to verify the injection site. Only data from rats with correction injection site were used

To observe the impact of sleep deprivation on fear memory and BDNF/ tropomyosin receptor kinase B (TrkB) pathway activity in the basal forebrain (see Figure 1, Experiment 1). Thirty‐three rats were randomly divided into two groups (*n* = 18 for each group) depending on whether subjected to sleep deprivation (*SD*) or not (RC). Immediately after sleep deprivation or control, fear training in the step‐down inhibitory avoidance test was initiated. Short‐term memory (STM) and long‐term memory (LTM) were then assessed 1 hr and 24 hr after training, respectively (*n* = 10 per group). To determine the expression of brain‐derived neurotrophic factor (BDNF) in the basal forebrain, the other 16 rats were euthanized and decapitated to collect the basal forebrain for Western blot (*n* = 4 per group) and immunohistochemical staining (*n* = 4 per group) one hour after behavior training just before the STM test.

#### 2.4.2 Experiment 2

To observe whether modulation of the BDNF/TrkB pathway in the basal forebrain could rescue the deficit of fear memory caused by sleep deprivation (seeFigure 1, Experiment 2). BDNF (#3897, CST) or ANA‐12 (S7745, Sellect.cn) was used to activate or inhibit basal forebrain TrkB signaling, respectively, before inhibitory avoidance training. Artificial cerebrospinal fluid (aCSF) was used as vehicle control for BDNF and ANA‐12. Eighty‐four rats were randomly divided into the six groups (*n* = 14 per group) according to sleep deprivation/control and/or BDNF/ANA‐12 treatment. The six groups were rest control followed by drug vehicle microinjection (RC + aCSF), rest control followed by BDNF microinjection (RC + BDNF), rest control followed by ANA‐12 microinjection (RC + ANA‐12), sleep deprivation followed by drug vehicle microinjection (*SD* + aCSF), sleep deprivation followed by BDNF microinjection (*SD* + BDNF), and sleep deprivation followed by ANA‐12 microinjection (*SD* + ANA‐12). Animals underwent bilateral cannula implantation with the tip 1mm above the basal forebrain. The rats were allowed 10 days of recovery after surgery. Then, animals were subjected to sleep deprivation or not, which was followed by fear training. One hour after fear training, 24 rats were euthanized to collect basal forebrain samples for Western blot examination of BDNF/TrkB signaling activity. The left 60 rats were subjected to both STM and LTM examination. After LTM evaluation, the rats were sacrificed. Hematoxylin and Eosin staining of brain was performed to verify the injection.

### 2.5 Step‐down inhibitory avoidance test

The step‐down apparatus was a 50 × 25 × 25 cm plastic cubic box fitted with a front transparent wall. The floor of the equipment was made up of steel bars, and a rubber platform (2.5 × 7 × 25 cm) was fixed on the left side of the floor. During the training session, the rat was placed on the platform, facing toward the steel bars. The rats received 0.4 mA/2.0 s electric shock (Izquierdo et al., 1995; Roesler et al., 2000) when they stepped down and put all four paws onto the bars. After training, the animals were placed again on the platform. Latency of step‐down to the steel bars was considered as a measure of memory retention for the aversive stimulus. Short‐ and long‐term memories were evaluated 1 hr and 24 hr after training, respectively. The maximum observation time was 300 s.

### 2.6 Stereotaxic surgery

**Figure 2:**
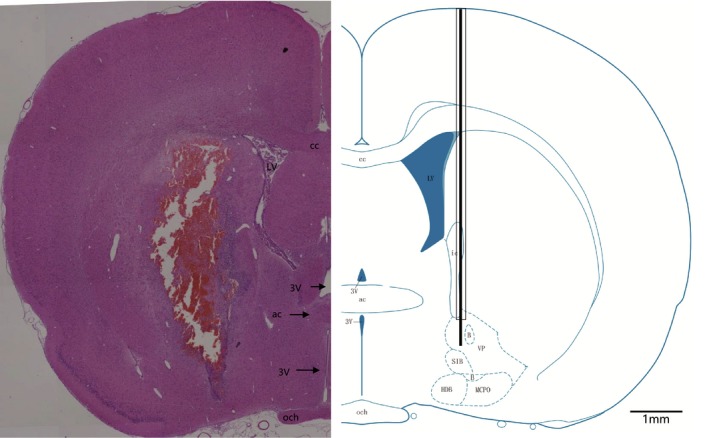
Microinjection probe tip location in the basal forebrain. A representative microinjection probe track of the coronal brain section is shown on the left, which is consistent with a schematic drawing of the coronal brain section of the 5th The Rat Brain In Stereotaxic Coordinates (Paxinos & Watson, 2005) on the right. MCPO, magnocellular preoptic area; HDB, nucleus of the horizontal limb of the diagonal band; SI, substantia innominate; och, optic chiasm; 3v, 3rd ventricle; ac, anterior commissure; cc, corpus callosum; LV, lateral ventricle; b, basal nucleus (Meynert); VP, ventral pallidum; ic, internal capsule

Rats were secured in a stereotaxic frame (RWD, China) under 2% isoflurane anesthesia, and surgical procedures were performed with further local infiltration of ropivacaine (0.5%, 1ml, AstraZeneca). The core body temperature of the rats was kept at around 37℃ with a thermostatic heating pad during surgery. The surgical area was disinfected, then 26‐gauge (OD = 0.46mm) guide cannulas (RWD, China) were inserted bilaterally at the area 1mm above the basal forebrain in 84 rats (*n* = 14 each group) (relative to Bregma: Anterior–Posterior (AP) −1.06 mm, Medial–Lateral (ML) ±2.57 mm, and Dorsal–Ventral (DV) −9 mm) and each cannula with a dummy cannula (30 gauge, OD = 0.31 mm) inserted was fixed on the skull with dental cement. Rats were single‐housed for 10 postoperative days. Schematic drawings of coronal brain sections from the 5th The Rat Brain In Stereotaxic Coordinates (Paxinos & Watson, 2005), and a digital photograph of a representative brain section with the probe track are shown in Figure 2.

### 2.7 Drug and vehicle control microinjections

BDNF (250ng/1µl/side; #3897; CST) was administrated ten minutes before behavior training (Valvassori et al., 2015). ANA‐12 (0.5 µg/1 µl/side; 7,745, Sellect.cn.) was administrated 1 hr before behavior training (Azogu & Plamondon, 2017; Shirayama et al., 2015). ANA‐12 is a selective TrkB receptor antagonist without altering TrkA and TrkC functions (Cazorla et al., 2011). The aCSF served as the vehicle control, which contained (in mM/l): 125 NaCl, 3.5 KCl, 26 NaHCO_3_, 1 MgCl_2_, and 10 glucose (pH = 7.4, 315 mOsm/L) (Kalemaki, Konstantoudaki, Tivodar, Sidiropoulou, & Karagogeos, 2018). The aCSF (1 µl/side) was administrated 1 hr before behavior training. Bilateral microinjection into the basal forebrain was delivered using a 10‐μL Hamilton syringe (Hamilton) with a microinfusion pump (0.5µl/min; Harvard Apparatus) (Schaich, Wellman, Koi, & Erdos, 2016). The drug was injected into the bilateral basal forebrain within 2 min. After injection, the syringe was indwelled for an additional 5 min to allow for diffusion. The injection sites were confirmed by visual inspection when the tissues were collected for Western blot or HE staining of the brain slices after the behavioral tests. Only data from rats with the correct injection sites were used for data analysis.

### 2.8 Tissue collection

The basal forebrain tissue was freshly obtained on ice according to reports from a previous study (Basheer, Porkka‐Heiskanen, Stenberg, & McCarley, 1999). Briefly, a 1‐mm‐thick coronal slice of brain was obtained by cutting in front of and behind the optic chiasm. Then, a horizontal cut was made on the coronal slice through the middle between the anterior commissure and the bottom of slice. Finally, the slice was cut vertically 1 mm lateral to the third ventricle and the middle of the olfactory tubercle on each side. The 1 mm × 1 mm × 2 mm basal forebrain tissue from each side obtained contains the magnocellular preoptic area (MCPO), the nucleus of the horizontal limb of the diagonal band (HDB), the substantia innominata (SI), and the basal nucleus (Basheer et al., 1999). Tissues were stored in a freezer at −80°C for further tests.

### 2.9 Immunohistochemistry

**Figure 3:**
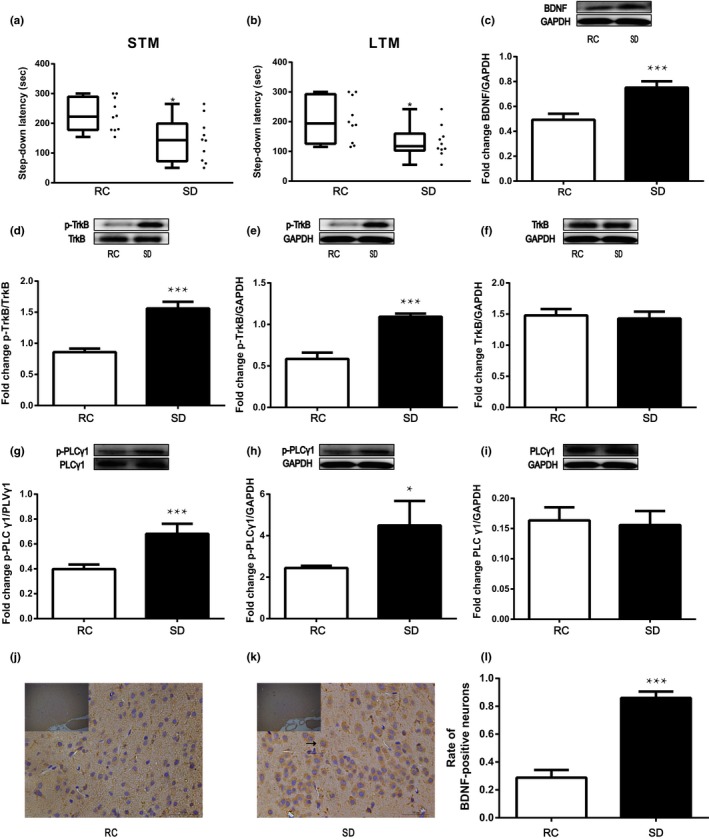
Fear memory consolidation impairments and BDNF/TrkB/PLCγ1 signaling activation following sleep deprivation. STM and LTM fear memories were evaluated using a step‐down apparatus and were shown impaired by 6 hr of sleep deprivation (*SD*). (a) Short‐term memory (STM) was tested an hour after training. (b) Long‐term memory (LTM) was tested 24 hr later. Data are expressed as box and whiskers (min to max) combined with scatter plot. ^*^, *p* < .05, compared to the rest control (RC) group; *n* = 10 per group. (c–i) Acute sleep deprivation activates BDNF/TrkB pathway signaling in basal forebrain. ^*^, *p* < .05 and ^***^, *p* < .001 versus RC. (j‐l) Immunostaining revealed that BDNF expression was increased in the basal forebrain neurons after sleep deprivation. A representative BDNF‐positive neuron is marked by the arrow (k). ^***^, *p* < .001 versus RC; *n* = 4 for each experimental group. Magnification, ×200. Bar = 50μm. Data are shown as mean + standard deviation (*SD*)

Immunohistochemistry was performed to investigate the BDNF expression changes in the basal forebrain after 6‐hr sleep deprivation. The brains of the rats were collected and fixed in 4% paraformaldehyde in PBS, and then, 5 μm of coronal sections were obtained from the paraffin block by cutting through the optic chiasm. Sections were blocked in Tris‐buffered saline with 0.1% Tween 20 (TBST) containing 5% goat serum for 1 hr at room temperature and then incubated overnight at 4°C with the rabbit anti‐BDNF (1:250, Abcam Cat#, ab108319, RRID: AB_10862052, Abcam). Sections were then washed three times and incubated at room temperature with biotinylated goat anti‐rabbit HRP‐conjugated secondary antibody IgG‐HRP (1:1,000, Abcam Cat# ab6721, RRID: AB_955447). The mounted slides were photographed using cell imaging observation equipment (LEICA DMI8, LEICA). An example of positive staining is shown in Figure 3f. Cases were scored as negatives if there was no or only weak BDNF staining. The nuclei were stained with hematoxylin. Four sections were obtained from each rat (*n* = 4 each group) and four visual fields were randomly selected and observed for each tissue section. The cells were counted, and the mean rates of positive cells from each rat were compared between the rest control and sleep deprivation groups.

### 2.10 Western blot

The proteins of the basal forebrain were extracted with a protein extraction kit (C500007, Sangon Biotech). The protein concentrations were measured using a BCA protein quantification kit (C503051, Sangon Biotech). About 30μg of protein from each sample were separated in 12% SDS/PAGE and then transferred onto polyvinyl difluoride membranes (10600023, GE, US) for 2h at 18V. After block, the membranes were incubated overnight at 4℃ with rabbit anti‐BDNF antibody (1:2000, Abcam Cat# ab108319, RRID:AB_10862052), anti‐phosphorylated TrkB (p‐TrkB) (1:1000, Millipore Cat# ABN1381, RRID:AB_2721199), anti‐TrkB (1:2000, Cell Signaling Technology Cat# 4603, RRID:AB_2155125), anti‐phosphorylated phospholipase C‐γ1 (p‐PLCγ1) (1:1,000, Abcam Cat# ab76155, RRID:AB_1310574), anti‐PLCγ1 (1:2000, Abcam Cat# ab90718, RRID:AB_10714566), or anti‐GAPDH (1:20000, Abcam Cat# ab181603, RRID:AB_2687666). On the following day, membranes were washed and then incubated for 1.5h at room temperature in Tris‐buffered saline with 0.1% Tween 20 (TBST) containing HRP‐conjugated secondary antibody IgG‐HRP (1:20000, Abcam Cat# ab6721, RRID: AB_955447). Finally, the blots were visualized with an electrochemiluminescence (ECL) kit (GE) and photographed using the Bio‐Rad imaging system. The optical density of the bands was determined using Quantity One software (http://www.bio-rad.com/zh-cn/SearchResults?Text=Quantity+One Quantity One 1‐D Analysis Software, RRID:SCR_014280). The protein densities of BDNF, p‐TrkB, TrkB, p‐PLCγ1, and PLCγ1 were normalized to GAPDH, the protein density of p‐TrkB or p‐PLCγ1 was normalized to TrkB or PLCγ1, respectively, and relative protein expressions were compared among groups.

### 2.11 Statistical analysis

The SPSS 18.0 software (https://www.ibm.com/products/spss-statistics SPSS, RRID:SCR_002865) was used for statistical analysis. As the behavioral test data did not follow a normal distribution, the data were expressed as median and interquartile combined with scatter dot diagrams. The behavioral data were further ranked, and the ranks were analyzed using one‐way analysis of variance (ANOVA) with post hoc Bonferroni pairwise comparison. Western blot and immunohistochemistry data were expressed as mean＋standard deviation (*SD*). One‐way ANOVA with post hoc Bonferroni's test was used for pairwise comparison. Statistical significance was set at *p* < .05 (two‐sided).

## 3 RESULTS

### 3.1 Acute sleep deprivation impairs both short‐ and long‐term fear memory

Figure 3a showed the short‐term memory evaluated as the latency. The latency was significantly decreased after sleep deprivation (*F*_1,18_ = 8.019,* p* = .011) compared with rest control. The latency measured 24 hr after the training was also decreased by sleep deprivation as compared with control (*F*_1,18_ = 7.007, *p* = .016, Figure 3b).

### 3.2 Acute sleep deprivation activates basal forebrain BDNF/TrkB signaling

After 6‐hr total sleep deprivation, brain‐derived neurotrophic factor (BDNF) protein expression in the basal forebrain was significantly increased (*F*_1,6_ = 81.512, 167% of control, *p* < .001 compared with control, Figure 3c). The increase in BDNF content was also confirmed by immunohistochemistry staining. The proportion of BDNF‐positive neurons was significantly increased after sleep deprivation (*F*_1,6_ = 257.484, *p* < .001, Figure 3j–l). The downstream tropomyosin receptor kinase B (TrkB) receptor phosphorylation (*F*_1,6_ = 174.770) and phospholipase C‐γ1 (PLCγ1) phosphorylation (*F*_1,6_ = 60.978) was also increased by sleep deprivation (both *p* < .05 compared with control, Figure 3d,e,g, and h). The TrkB (*F*_1,6_ = 0.789) and PLCγ1 (*F*_1,6_ = 0.323) protein levels (Figure 3f,i) were not significantly different between sleep deprivation and control groups (both *p* > .05).

### 3.3 Microinjection of BDNF into the basal forebrain rescued fear memory deficit by sleep deprivation

**Figure 4:**
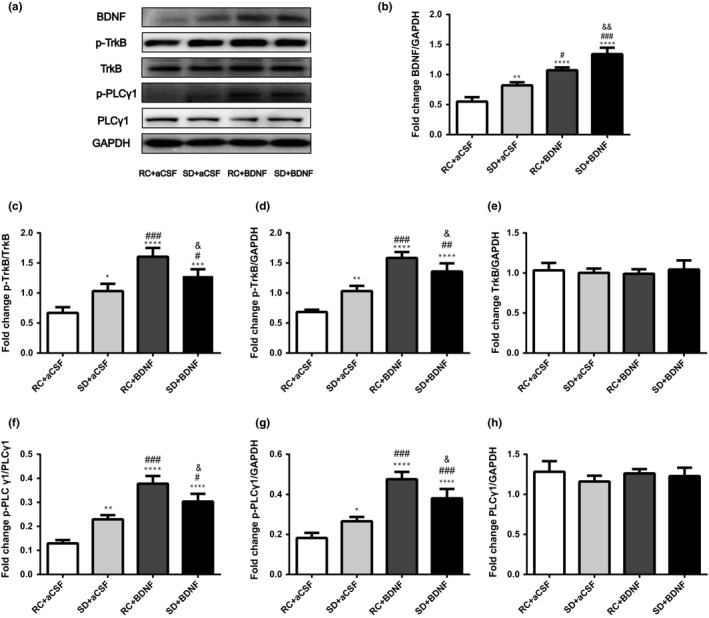
Effects of bilateral microinjection of BDNF (250 ng/1 µl/side) into the basal forebrain on BDNF/TrkB/PLCγ1 signaling. Rats in the rest control (RC) and sleep deprivation (*SD*) groups received artificial cerebrospinal fluid (aCSF, vehicle) or BDNF microinjection. One hour after fear training, rats were euthanized to collect basal forebrain samples for Western blot examination of BDNF/TrkB signaling activity. (a) A representative Western blot image shows bands of BDNF/TrkB proteins and loading control GAPDH. (b) The relative protein expression of BDNF, (C AND D) p‐TrkB, (e) TrkB, (f and g) p‐PLCγ1, and (h) PLCγ1. ^*^, *p* < .05; ^**^, *p* < .01; ^***^, *p* < .001 and ^****^, *p* < .0001 compared to the RC + aCSF group. ^#^, *p* < .05; ^##^, *p* < .01 and ^###^, *p* < .001 compared to the *SD* + aCSF group. ^&^, *p* < .05 and ^&&^, *p* < .01 compared to the RC + BDNF group. All data were expressed as mean + standard deviation (*SD*) (*n* = 4 per group)

BDNF contents in the basal forebrain were significantly increased after BDNF microinjection (*F*_3,12_ = 90.893, Figure 4a,b). The downstream phosphorylation of TrkB (*F*_3,12_ = 55.305) and PLCγ1 (*F*_3,12_ = 66.886) were also increased by BDNF in both control and sleep deprivation group (all *p* < .05, Figure 4a,c,d,f,g). TrkB (*F*_3,12_ = 0.351) and PLCγ1 (*F*_3,12_ = 1.776) protein levels in the basal forebrain were not significantly different among the four groups (both *p* > .05, Figure 4a,e,h).

**Figure 5:**
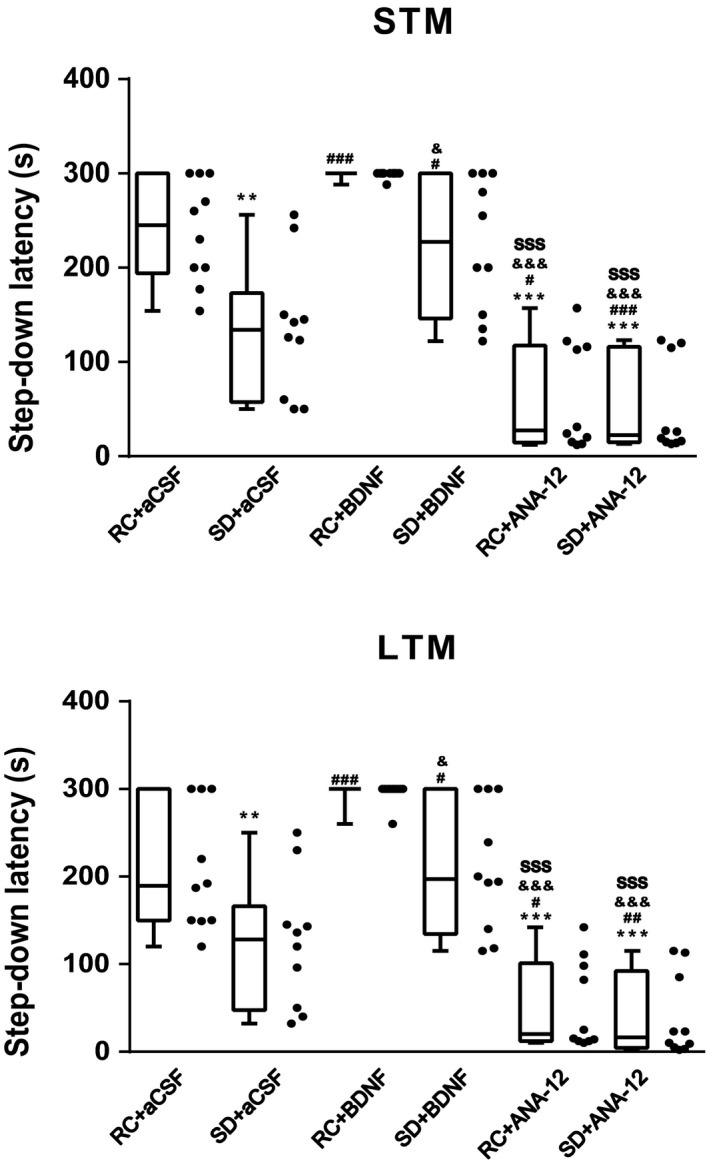
Effects of bilateral microinjection of BDNF (250 ng/1 µl/side) or ANA‐12 (0.5 µg/1 µl/side) into the basal forebrain on sleep deprivation (*SD*)‐induced fear memory impairments. Short‐term memory (STM) was tested an hour after training session. Long‐term memory (LTM) was tested 24 hr later. Data (*n* = 10 in each group) were expressed as box and whiskers (min to max) combined with scatter plot. ^**^, *p* < .01 and ^***^, *p* < .001 compared to the rest control (RC) +artificial cerebrospinal fluid (aCSF) group. ^#^, *p* < .05; ^##^, *p* < .01 and ^###^, *p* < .001 compared to *SD* + aCSF group. ^&^, *p* < .05 and ^&&&^, *p* < .001 compared to RC + BDNF group. ^sss^, *p* < .001 compared to *SD* + BDNF group

We next investigated whether activation of the BDNF/TrkB pathway by BDNF was correlated with memory enhancement (Figure 5). After BDNF microinjection, the latency measured 1 hr (*F*_5,54_ = 37.081, *p* < .001) and 24 hr (*F*_5,54_ = 37.029, *p* < .001) after fear training was significantly prolonged in the sleep deprivation group. For the rest control rats, exogenous BDNF caused no differences in the short‐ and long‐term memory performance.

### 3.4 TrkB receptor antagonist ANA‐12 aggravated sleep deprivation‐induced fear memory impairments

**Figure 6:**
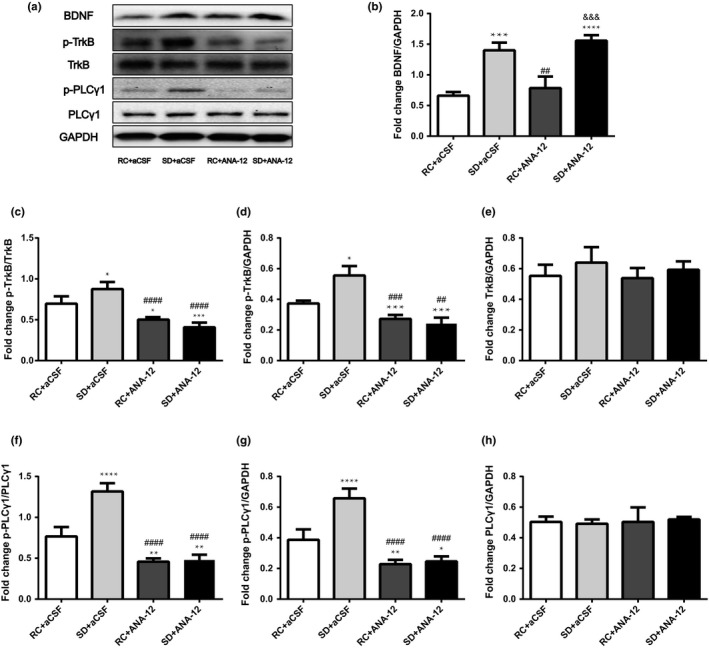
Effects of bilateral basal forebrain microinjection of TrkB receptor antagonist ANA‐12 (0.5 μg/1 µl/side) on sleep deprivation (*SD*)‐induced BDNF/TrkB/PLCγ1 activation. ANA‐12 bilateral microinjection into the basal forebrain caused down‐regulation of the BDNF/TrkB pathway. Rats in the rest control (RC) and sleep deprivation groups received artificial cerebrospinal fluid (aCSF) or ANA‐12 microinjection. One hour after fear training, rats were euthanized to collect basal forebrain samples for Western blot examination of BDNF/TrkB signaling activity. (a) A representative Western blot shows bands of BDNF/TrkB proteins and loading control GAPDH. (b) The relative protein expression of BDNF, (c, d) p‐TrkB, (e) TrkB, (f, g) p‐PLCγ1, and (h) PLCγ1. ^*^, *p* < .05; ^**^, *p* < .01; ^***^, *p* < .001 and ^****^, *p* < .0001, compared to the RC + aCSF group. ^##^, *p* < .01; ^###^, *p* < .001 and ^####^, *p* < .0001 compared to the *SD* + aCSF group. ^&&&^, *p* < .001 compared to the RC + ANA‐12 group. All data were expressed as mean + standard deviation (*SD*) (*n* = 4 per group)

The Western blot results showed that TrkB phosphorylation in the basal forebrain (Figure 6a,c–e) was significantly decreased by ANA‐12 in both the rest control and sleep deprivation groups (*F*_3,12_ = 35.485, both *p* < .05). Downstream p‐PLCγ1 levels were also reduced by ANA‐12 in both the rest control and sleep deprivation groups (*F*_3,12_ = 88.035, all *p* < .05, Figure 6a,f–h). The increase in BDNF expression induced by sleep deprivation was not changed by ANA‐12 (*p* > .05, Figure 6a,b). In the step‐down avoidance test, we found ANA‐12 induced both short‐term and long‐term memory impairment in the rest control group as supported by shortened latency compared with vehicle (Figure 5). ANA‐12 injection after sleep deprivation further reduced the latency measured 1 hr and 24 hr after fear training.

## 4 DISCUSSION

We found that 6‐hr total sleep deprivation‐induced impairments of fear memory were accompanied with increased brain‐derived neurotrophic factor (BDNF) protein levels in the basal forebrain. Subsequently, we injected exogenous BDNF or tropomyosin receptor kinase B (TrkB) antagonist (ANA‐12) into the basal forebrain of rats before inhibitory avoidance training. The results showed that bilateral microinjection of BDNF activated the BDNF/TrkB pathway and partly reversed short‐ and long‐term impairments in fear memory induced by acute sleep deprivation (Figure 5). Moreover, the selective TrkB receptor antagonists ANA‐12 inhibited the downstream phosphorylation of TrkB (Y816) and phospholipase C‐γ (PLCγ) (Y783) and aggravated sleep deprivation‐induced fear memory deficits. Taken together, these results supported our hypothesis that enhanced basal forebrain BDNF/TrkB signaling acted as a compensation to counteract fear memory impairments caused by sleep deprivation.

Expression of BDNF has been shown to be related to the sleep debt of rats. For example, the levels of BDNF in the cerebral cortex rise with extended wakefulness (Cirelli & Tononi, 2000). The increase of BDNF after sleep deprivation in this research (Figure 3c) were consistent with human studies and rat studies following sleep deprivation protocols (Giacobbo et al., 2016; Wallingford, Deurveilher, Currie, Fawcett, & Semba, 2014). Increase in BDNF protein level could be caused by enhanced expression and secretion, or reduced degradation of BDNF (Lessmann & Brigadski, 2009). After acute sleep deprivation, an exon‐specific increase in the expression of BDNF transcripts 1, 4, and 9a was found in the basal forebrain, while the 5‐methylcytosine DNA modification, which is important for the daily regulation of BDNF in the basal forebrain, was absent (Ventskovska, Porkka‐Heiskanen, & Karpova, 2015). Based on these results, the increased expression of BDNF might be attributed to increased transcription and translation. It is tempting to relate the preservation of fear memory with the increased BDNF levels seen in the sleep deprived rats. Indeed, brain adaptive response after acute sleep deprivation has been confirmed in gene expression (Cirelli, Gutierrez, & Tononi, 2004), neurotransmitter release (Dash, Douglas, Vyazovskiy, Cirelli, & Tononi, 2009), and compensatory recruitment of different brain structures, such as hippocampus (Yan et al., 2019). In this context, and considering the pivotal functions of BDNF in memory consolidation, elevation of transcription of BDNF during acute sleep deprivation could play a modulating role of the fear memory process.

BDNF could activate three main signaling pathway through binding to the TrkB receptor. The Ras/mitogen‐activated protein kinase (MAPK) pathway and the phosphoinositide 3‐kinase (PI3K) pathway are activated primarily through Shc/FRS‐2 binding to Y515, whereas the PLCγ pathway is activated through Y816 phosphorylation (Minichiello et al., 2002). Among them, BDNF/TrkB (Y816)/ PLCγ (Y783) activation was necessary and sufficient to mediate synaptic plasticity and was involved in short‐ and long‐term fear memory formation (Alonso et al., 2005; Minichiello et al., 2002). Our results found that increased basal forebrain BDNF expression was accompanied with enhanced activation of TrkB (Y816)/ PLCγ (Y783), implicating a role of this signaling pathway in sleep deprivation‐induced fear memory impairments. We further used ANA‐12, a highly potent and selective TrkB inhibitor to testify this. We found that injection of ANA‐12 abolished the increase in p‐TrkB and p‐PLCγ1 levels induced by sleep deprivation which were consistent with results from recent study (Contreras‐Zárate et al., 2019). Furthermore, ANA‐12 aggravated sleep deprivation‐induced memory impairments (Figure 5). These results suggested BDNF/TrkB (Y816)/ PLCγ (Y783) activation as a compensatory way to reduce the adverse effects of sleep deprivation on memory.

Of note, ANA‐12 inhibits both two phosphorylation sites (Y515/Shc site; Y816/PLCγ site) and downstream processes of TrkB (Cazorla et al., 2010, 2011). It has been found that the PLCγ docking site, but not the Shc docking site, is necessary to mediate the TrkB‐dependent acquisition of fear in hippocampus and amygdala (Minichiello et al., 2002; Musumeci et al., 2009). We also found that ANA‐12 inhibited both fear memory and PLCγ phosphorylation (Figures 5 and 6f,g). Taken together, these results suggested TrkB Y816/PLCγ signaling but not Y515/Shc signaling was involved in fear memory acquisition and retention. However, there is still a need to clarify whether Y515/Shc signaling pathway in basal forebrain is involved in sleep deprivation‐induced fear memory impairments.

It was previously suggested that stimulation with BDNF could result in a very robust phosphorylation signal of TrkB and PLCγ in basal forebrain cholinergic neurons (Knüsel, Rabin, Widmer, Hefti, & Kaplan, 1992). The result was consistent with our findings (Figure 4). Interestingly, the enhanced BDNF expression could also activate basal forebrain cholinergic neurons (Nonomura & Hatanaka, 1992) and then resulted in an increase of BDNF mRNA expression in the hippocampus (Boatell et al., 1992). Moreover, rescue of impaired cAMP signaling after short‐term sleep deprivation could enhance BDNF expression in the hippocampus and mitigate sleep deprivation‐induced fear memory impairments (Vecsey et al., 2009) (Guo et al., 2016). Considering that the main subcortical input of hippocampus is from the medial septum of the basal forebrain cholinergic system (Solari & Hangya 2018) and the septo‐hippocampal pathway, the main projection of medial septal neurons (Everitt & Robbins, 1997), is also important for processing of aversive information during fear conditioning (Calandreau, Jaffard, & Desmedt, 2007; Stepanichev, Lazareva, Tukhbatova, Salozhin, & Gulyaeva, 2014), there might be a possibility that sleep deprivation‐induced increase in basal forebrain BDNF might activate basal forebrain cholinergic neurons which further increased hippocampal BDNF expression and fear memory consolidation. Although recent research showed BDNF level in hippocampus was elevated by 12‐hr sleep deprivation (Yan et al., 2019), whether BDNF/TrkB (Y816)/ PLCγ(Y783) signaling in hippocampus was activated after sleep deprivation needs further confirmation.

The 75‐kDa neurotrophin receptor (p75^NTR^) is another transmembrane receptor for BDNF. P75^NTR^ is a low‐affinity receptor for BDNF but a high‐affinity for precursor of BDNF (proBDNF) (Matsumoto et al., 2008; Woo et al., 2005; Yang et al., 2009). Interestingly, BDNF and proBDNF play distinct roles in fear memory processing (Lu, Pang, & Woo, 2005). The administration of cleavage‐resistant proBDNF or its antibody into the medial prefrontal cortex (mPFC) could facilitate or block fear extinction (Sun, Li, & An, 2018). Further studies are needed to investigate the role of basal forebrain P75^NTR^ signaling in sleep deprivation‐induced memory deficits.

## 5 CONCLUSION

Acute sleep deprivation induces compensatory increase of BDNF expression and activation of TrkB/PLCγ1 signaling in the basal forebrain. Microinjection of BDNF into the basal forebrain mitigates the fear memory impairments caused by sleep deprivation by activating TrkB/PLCγ1 signaling.

## CONFLICT OF INTEREST

The authors have no conflict of interest to declare. The authors are willing to meet costs of color reproduction if needed.

## AUTHOR CONTRIBUTION

Wei‐Dong Mi designed the research, prepared the manuscript, and made the revision. Tao Ma and Hao Zhang participated in the whole set of experiments and wrote the manuscript. Zhi‐Peng Xu, Yan Lu, and Qiang Fu conducted the animal behavioral tests. Wei Wang, Guan‐Hua Li, and Ying‐Ying Wang conducted the Western blot experiments. Yi‐Tian Yang checked the data and made the statistical analysis. Tao Ma and Hao Zhang contributed equally to this work.

## Data Availability

The data used during the study are available from the corresponding author by request.
